# Maturation of bovine cumulus oocyte complexes in follicular fluid with or without estradiol, progesterone or the combination affects cumulus cell expansion and blastocyst development

**DOI:** 10.1371/journal.pone.0321266

**Published:** 2025-06-02

**Authors:** Audra W. Harl, Verónica M. Negrón-Pérez, Jacob W. Stewart, George A. Perry, Alan D. Ealy, Michelle L. Rhoads

**Affiliations:** 1 School of Animal Sciences, Virginia Tech, Blacksburg, Virginia, United States of America; 2 Texas A&M AgriLife Research and Extension Center, Overton, Texas, United States of America; Justus Liebig Universitat Giessen, Germany

## Abstract

Although laboratory procedures for *in vitro* bovine embryo production have improved immensely, developmental capacity following fertilization is still limited, especially in comparison to *in vivo*-produced embryos. *In vivo*, the maturing oocyte is enclosed in the ovarian follicle and surrounded by its cumulus cells and follicular fluid. Hormones and other components of the follicular fluid change dynamically as the follicle develops and approaches ovulation. The importance of the *in vivo* follicular microenvironment for oocyte developmental competence has not been well defined, however. Therefore, the objective of this study was to investigate the impact of follicle size and relative estradiol and progesterone concentrations on cumulus cell expansion and early embryo development following follicular fluid exposure during maturation *in vitro*. All experiments and replicates contained a standard formulation control maturation medium (cOMM). Follicular fluid was collected via needle aspiration from small (2–5 mm diameter) and large (10–20 mm diameter) follicles and pooled according to size. The follicular fluid was added to a hormone-free base medium (eOMM) as follows: supplemented with 75% untreated large follicular fluid (LFF75), 75% untreated small follicular fluid (SFF75), 75% charcoal-stripped large follicular fluid (csLFF75), 75% charcoal-stripped small follicular fluid (csSFF75). Progesterone and/or estradiol were added to the charcoal-stripped follicular fluid treatments based on average concentrations found in fluid from pooled large or pooled small follicles. These six treatment media were formulated using eOMM as a base with the following designations and additions: 75% charcoal-stripped large follicular fluid + 37 ng/ml estradiol (csLFF+E2), 75% charcoal-stripped small follicular fluid + 23 ng/ml estradiol (csSFF+E2), 75% charcoal-stripped large follicular fluid + 160 ng/ml progesterone (csLFF+P4), 75% charcoal-stripped small follicular fluid + 140 ng/ml progesterone (csSFF+P4), 75% charcoal-stripped large follicular fluid + 37 ng/ml estradiol + 160 ng/ml progesterone (csLFF+E2+P4), or 75% charcoal-stripped small follicular fluid + 23 ng/ml estradiol + 140 ng/ml progesterone (csSFF+E2+P4). Cumulus expansion in the csSFF75 maturation medium was less than that of its untreated counterpart (SFF75), while cumulus cell expansion was similar for LFF75 and csLFF75. The addition of estradiol to the follicular fluid treatments was beneficial and improved cumulus cell expansion to values similar to cOMM, while progesterone alone had no effect. The greatest cumulus cell expansion was observed when both estradiol and progesterone were added to the follicular fluid treatments. Cleavage rates were generally reduced by follicular fluid treatments, with the exception being csSFF+P4 which had a cleavage rate similar to oocytes matured in cOMM. Blastocyst rates for LFF75 and csLFF75 were similar to cOMM, while SFF75 and csSFF75 reduced blastocyst rates. Interestingly, the follicular fluid treatments with added progesterone either maintained or improved blastocyst rates such that csLFF+P4 and csSFF+P4 were similar to cOMM. The same could not be said for any of the treatments containing estradiol, whether alone or in combination with progesterone. Taken together, these results suggest a dichotomous relationship between estradiol and progesterone during maturation. Estradiol supports cumulus cell expansion while progesterone concentrations during oocyte maturation are more important for subsequent embryo development.

## Introduction

Over the decades, laboratory procedures and conditions for *in vitro* fertilization and embryo culture have evolved in order to maximize embryo production. Despite the progress that has been made, developmental capacity following fertilization is limited at best, with only around one-third of bovine oocytes placed into maturation resulting in viable embryos [1]. The low efficiency of bovine embryo production *in vitro* is a multifaceted problem, with factors like oocyte quality, media composition, and culture environment all playing major roles in the successful (or unsuccessful) production of embryos [2]. In an effort to improve *in vitro* embryo production, researchers have closely examined and attempted to recreate aspects of the natural *in vivo* follicular environment. During *in vivo* development, final maturation of the bovine oocyte takes place within the dominant follicle under the influence of gonadotropins, steroids, and growth factors [2, 3]. In the preovulatory stage, follicular components change as ovulation approaches, and it is likely that many important components and interactions have yet to be identified. Considering the complexity of the environment in which oocytes and early embryos develop *in vivo*, it is in some ways astounding that viable embryos can be produced successfully *in vitro*.

Fortunately, the events stimulated by the preovulatory LH surge *in vivo* can be replicated *in vitro* sufficiently well for embryo production, albeit at comparatively low rates. Manual removal of the bovine cumulus oocyte complex (COC) from the follicle results in spontaneous meiotic maturation and cumulus cell expansion, thus facilitating *in vitro* maturation of oocytes in preparation for fertilization [1]. The spontaneous resumption of meiosis following removal of the bovine COC from the follicle was first demonstrated in 1935, although the mechanism(s) responsible remains unclear [4]. Irrespective of the mechanism(s) however, *in vitro* bovine oocyte nuclear maturation rates are high, frequently exceeding 80% [5–7], and previous research has shown that cumulus cell expansion is important for subsequent successful fertilization [8, 9].

Even though the nuclear maturation of the bovine oocyte is generally successful under standard *in vitro* conditions, usually less than half of successfully matured oocytes will go on to develop to the blastocyst stage. This indicates there are other developmental hurdles that must be overcome for maximal production of viable embryos. *In vivo*, the majority, if not all of these hurdles are overcome prior to follicle rupture while the COC is contained within the follicular microenvironment. An important component of this microenvironment is the follicular fluid, and it has been suggested that the addition of follicular fluid to *in vitro* media during maturation could improve developmental results [10]. The addition of follicular fluid to maturation media has produced mixed results in the bovine, with some reporting positive, neutral and/or negative effects on aspects of oocyte maturation and early embryo development [1, 11–19]. Unfortunately, comparison of results between studies is complicated by important differences in experimental designs that directly impact the outcomes. Examples of these differences include, but are not limited to the status of the animal from which the follicular fluid is collected, the stage of the follicle from which the follicular fluid is collected, the concentration of follicular fluid in the maturation media and whether or not exogenous hormones are added to the maturation media.

Despite the challenges associated with interpreting differences between studies, there are some points of commonality. The most consistently reported findings are that the addition of follicular fluid to maturation media increases cumulus cell expansion, but this improvement in expansion does not automatically equate to an improvement in subsequent blastocyst rates [12, 16, 19]. Most studies have also found that maturation of COCs in follicular fluid concentrations >50% is generally detrimental for subsequent blastocyst rates [13, 14, 19]. Another point of agreement amongst researchers is that the components within the follicular fluid that are responsible for the observed effects have not been fully characterized. Indicators of important factors in follicular fluid can be gleaned from the results of previous work, however. In experiments where fluids from different sized follicles are tested side-by-side, maturation in follicular fluid from larger follicles promotes blastocyst development over the fluid from smaller follicles [1, 17, 19], indicating that hormone concentrations within the follicular fluid contribute to the processes of oocyte maturation in preparation for fertilization and early embryonic development. This is further supported by the fact that the source of follicular fluid (animal status and stage of follicle development) affects oocyte- and embryo-related outcomes across different experiments, as hormone concentrations would vary accordingly.

Follicular fluid contains a myriad of hormones, any or all of which could be regulating oocyte maturation and developmental competence alone or in concert with other hormones, cytokines or growth factors. Of particular interest for oocyte maturation are the steroid hormones, the effects of which have been studied for decades [3, 20, 21]. Estradiol and progesterone are of particular interest because they change dynamically in the follicular fluid as the follicle develops to the preovulatory stage and are known to play important roles in oocyte maturation [20]. Thus, it is likely that their relative concentrations are influential when follicular fluid is added to *in vitro* maturation media. As such, the objective of this study was to investigate the impact of relative estradiol and progesterone concentrations in follicular fluid from different sized follicles on an aspect of oocyte maturation (cumulus cell expansion) and subsequent embryo development. We hypothesized that the inherent differences in estradiol and progesterone concentrations between the small and large follicular fluid are a major factor contributing to observed differences in embryo development following exposure to those fluids during *in vitro* oocyte maturation.

## Materials and methods

All bovine IVF and IVC protocols were based on previously described procedures [22].

### Collection and preparation of follicular fluid

Bovine ovaries were collected from an abattoir (Brown Packing, Gaffney, SC) and transported to the laboratory in 0.9% saline supplemented with penicillin (100 units/ml) and streptomycin (100 units/ml). Follicular fluid was collected via needle aspiration from small (2–5 mm diameter) and large (10–20 mm diameter) follicles and pooled according to size into a 15 ml conical tube. Size designations for small and large follicles were based upon previous publications [23–26]. Analyses used only small and large follicular fluid (not medium follicular fluid) because those sizes represent the extremes and are physiologically relevant. When oocytes are collected for IVF, they are collected from small follicles (and thus exposed to small follicular fluid). When oocytes are ovulated naturally *in vivo*, they ovulate from large follicles (and thus exposed to large follicular fluid). Fluid was filtered through a 0.2 μm cell strainer into a 15 ml conical tube, then stored at -20 °C. After several replicates of follicular fluid collections, follicular fluid was thawed, brought to room temperature and pooled. Half of each type of follicular fluid (from large or small follicles) was separated into a 15 ml conical tube for charcoal stripping in order to remove the majority of the steroid hormones as previously described [27, 28]. Briefly, follicular fluid was filtered through a 0.2 μm cell strainer to remove debris. Activated charcoal (Sigma-Aldrich, St. Louis, MO, USA) was added to follicular fluid at a rate of 10 mg/ml, then incubated on a shaker at low-medium speed at room temperature for 1 h. The fluid-charcoal mixture was then centrifuged at 4 °C for 20 minutes at 10,000 x g. Fluid was then decanted into aliquots of 1ml and frozen at -20 °C until use. Fetal bovine serum (FBS; Hyclone, Grand Island, NY, USA) was charcoal stripped according to the above protocol and stored in 5 ml aliquots at -20 °C until use.

### Progesterone and estradiol measurement and preparation

In order to determine progesterone and estradiol inclusion rates for subsequent experiments, each steroid was measured in pooled aliquots from small or large follicular fluid samples. The concentration of progesterone was measured in a single assay using a commercially available radioimmunoassay kit (Coat-A-Count, Siemens Medical Solutions Diagnostics, Los Angeles, CA, USA) [29] with an intra assay CV of 3.7%. Estradiol concentrations within the follicular fluid were also measured in a single assay by validated radioimmunoassay [30] with an intra assay CV of 4.74%.

Progesterone (Fisher Scientific AC225650050-5G) was diluted in pure ethanol at a rate of 1 mg/ml as per manufacturer recommendations, then diluted again into cell culture water at a rate of 1 ng/μl. Aliquots of 65 μl were stored at -20 °C until use. β-Estradiol (Sigma-Aldrich E2758-1G) was diluted in pure ethanol at a rate of 1 mg/ml as per manufacturer recommendations, then diluted again into cell culture water (Sigma-Aldrich W3500-500mL) at a rate of 1 ng/μl and frozen in 40 μl aliquots at -20 °C until use.

### Maturation media preparation

Table 1 contains a summary of all media treatments and abbreviations. Two control media were used in this experiment. One was designated the positive control oocyte maturation medium (cOMM) and served as an indicator of successful replicates. It was the usual formulation used in our laboratory and included untreated fetal bovine serum (FBS; not charcoal stripped; 21.79 pg/ml estradiol and 0.45 ng/ml progesterone in FBS). Specifically, this medium consisted of TCM-199+ Earls Salts (LifeTech 11150–059), 10% FBS (ThermoFisher 10437010), 1.14% Glutamax 100X (Gibco 35050–061), 1.14% sodium pyruvate (LifeTech 11360–070), 40 μg/ml follicle-stimulating hormone (FSH; Folltropin®, AgTech INC), estradiol (Sigma-Aldrich E2758-1G), 50 μg/ml gentamycin (Sigma-Aldrich), and EGF (Sigma-Aldrich, E9644-.5MG) and was not supplemented with any follicular fluid. Negative control medium (eOMM) consisted of TCM-199+ Earls Salts, 10% charcoal-stripped FBS, 1.14% Glutamax 100X, 50 µg/ml gentamicin (Gibco, Grand Island, NY, USA), and 1.14% sodium pyruvate and was not supplemented with any hormones or follicular fluid. The eOMM was a stand-alone treatment and also served as the base oocyte maturation medium (OMM) for all experimental treatments. Progesterone and/or estradiol were added to the charcoal-stripped follicular fluid treatments based on average concentrations found in fluid from pooled large or pooled small follicles (measurements described above). Thus, the eOMM was supplemented with one of the following: 75% untreated large follicular fluid (LFF75), 75% untreated small follicular fluid (SFF75), 75% charcoal-stripped large follicular fluid (csLFF75), 75% charcoal-stripped small follicular fluid (csSFF75), 75% charcoal-stripped large follicular fluid + 37 ng/ml estradiol (csLFF+E2), 75% charcoal-stripped small follicular fluid + 23 ng/ml estradiol (csSFF+E2), 75% charcoal-stripped large follicular fluid + 160 ng/ml progesterone (csLFF+P4), 75% charcoal-stripped small follicular fluid + 140 ng/ml progesterone (csSFF+P4), 75% charcoal-stripped large follicular fluid + 37 ng/ml estradiol + 160 ng/ml progesterone (csLFF+E2+P4), or 75% charcoal-stripped small follicular fluid + 23 ng/ml estradiol + 140 ng/ml progesterone (csSFF+E2+P4). As previously stated, standard cOMM and unsupplemented eOMM were used as control groups.

**Table 1:** Description of maturation media, including treatment abbreviation, rate of supplementation, hormone concentration, and size of follicle from which the fluid was collected.

Treatment	Contents
cOMM	Normal oocyte maturation medium (OMM)
eOMM	OMM with no estradiol or FSH added that has been charcoal stripped
LFF75	eOMM + 75% large follicular fluid
SFF75	eOMM + 75% small follicular fluid
csLFF75	eOMM + 75% charcoal stripped large follicular fluid
csSFF75	eOMM + 75% charcoal stripped small follicular fluid
csLFF+E2	eOMM + 75% csLFF75 + 37 ng/mL estradiol
csSFF+E2	eOMM + 75% csSFF75 + 23 ng/mL estradiol
csLFF+P4	eOMM + 75% csLFF75 + 160 ng/mL progesterone
csSFF+P4	eOMM + 75% csSFF75 + 140 ng/mL progesterone
csLFF+E2+P4	eOMM + 75% csLFF75 + 37 ng/mL estradiol + 160 ng/mL progesterone
csSFF+E2+P4	eOMM + 75% csSFF75 + 23 ng/mL estradiol + 140 ng/mL progesterone

### Experiment 1: Cumulus cell expansion following maturation with varying estradiol and progesterone concentrations in follicular fluid

For Experiment 1, the previously described charcoal–treated fluid was used. Some aliquots were supplemented with estradiol and/or progesterone to test whether estradiol and/or progesterone directly affects cumulus cell expansion.

Oocytes were collected from antral follicles (2–7 mm diameter) of abattoir-derived ovaries via slashing. Larger follicles were ruptured first so that their contents could be discarded. Follicle contents from follicles of the desired size were then collected into ~150 ml of oocyte collection medium (OCM) as previously described [31]. Medium was then filtered through a 0.2 μm cell strainer. The material collected by the filter was rinsed onto a gridded plate for search and collection of COCs. Cumulus oocyte complexes with homogeneous cytoplasm and more than three layers of cumulus cells were collected (n=480) and washed twice in fresh OCM. They were then randomly distributed into individual 10 µl drops of one of the maturation media described above, overlaid with mineral oil and matured for 19 h at 38.5°C in an atmosphere of 5% (v/v) CO_2_ in humidified air.

Digital images were acquired at time 0 h (when placed in maturation medium) and time 19 h using an inverted microscope (EVOS XL). The area of each COC was quantified from the images using ImageJ software v 1.47 (NIH). Then, COC expansion was calculated as percent increase in area for each individual COC. The COCs with an expansion percent increase lower than 10% were excluded from analyses. A total of four replicates were analyzed with 40 COCs per treatment.

### Experiment 2: Embryo development following oocyte maturation with varying estradiol and progesterone concentrations in follicular fluid

Cumulus-oocyte complexes (n=4,006; 16 replicates) were collected as described above and matured in groups of 15 in 50 μl drops of one of the control or treatment maturation media while overlaid with mineral oil (Origio, Målov Denmark) for 21 h at 38.5 °C under 5% CO_2_.

After being subjected to treatments during the maturation period, all COCs were treated the same for the remainder of the procedures. All *in vitro* fertilization and *in vitro* culture procedures were based on previously described protocols [22, 32]. Briefly, after 21 h maturation, COCs from the same treatment were pooled, washed three times in HEPES- Tyrode’s albumin lactate pyruvate [HEPES-TALP; HEPES-TL (Caisson Laboratories, Inc; North Logan, UT, USA) supplemented with 3 mg/ml BSA (Fraction V), 22 µg/ml sodium pyruvate and 75 µg/ml gentamicin] and fertilized in plates containing 500 µl of IVF-TALP [IVF-TL (Caisson Laboratories) supplemented with 6 mg/ml BSA (essentially fatty acid free), 22 µg/ml sodium pyruvate, 10 µg/ml heparin and 50 µg/ml gentamicin]. Two frozen-thawed semen straws from two *B. taurus* bulls were pooled, purified with BoviPure-BoviDilute 40% [v/v and 80% (v/v)], and diluted to a final concentration in the fertilization dishes of 1 x 10^6^/ml. Fertilization time was 18–22 h in a humidified gas atmosphere of 5% (v/v) CO_2_ and 19% (v/v) O_2_ at 38.5 °C for all groups.

Putative zygotes were collected, exposed to hyaluronidase (1000 U/ml in ~0.5 ml HEPES-TALP) and vortexed for 5 minutes to remove cumulus cells. Putative zygotes were then washed three times in HEPES-TALP and placed in groups of 15 zygotes per 25 µl drop of synthetic oviductal fluid – bovine embryo 2 (SOF-BE2) covered with mineral oil in a humidified gas atmosphere of 5% (v/v) CO_2_, 5% (v/v) O_2_ and the balance nitrogen, at 38.5 °C. Cleavage rates were assessed on day 3 and blastocyst rates at day 8 post-fertilization. A replicate was defined as the COCs collected in one day for *in vitro* fertilization procedures and consisted of either 30 or 60 oocytes per treatment. Only replicates with a cleavage rate of ≥65% in COMM embryos were included in data analysis.

### Statistical analysis

The SAS v 9.4 software package (SAS Institute Inc., Cary, NC, USA) was used for statistical analysis. Data were analyzed for the main effect of treatment, replicate and their interaction using PROC MIXED and PROC GENMOD procedures of SAS. The dependent variables included COC expansion, cleavage of embryos and development to blastocysts. Cumulus oocyte expansion was calculated as a percentage increase in area from the beginning to end of the maturation period. Day 3 cleavage rate was calculated as the number of cleaved embryos divided by the total number of oocytes and d 8 blastocyst rate was calculated as the total number of blastocysts divided by the total number cleaved. Separation of means was conducted with the LSMEANS statement in SAS with the Tukey adjustment. Results are reported as least squares means ± standard error of the mean. Statistical significance was declared at P<0.05.

## Results

### Experiment 1: Cumulus cell expansion following maturation with varying estradiol and progesterone-containing follicular fluid

**Fig 1:**
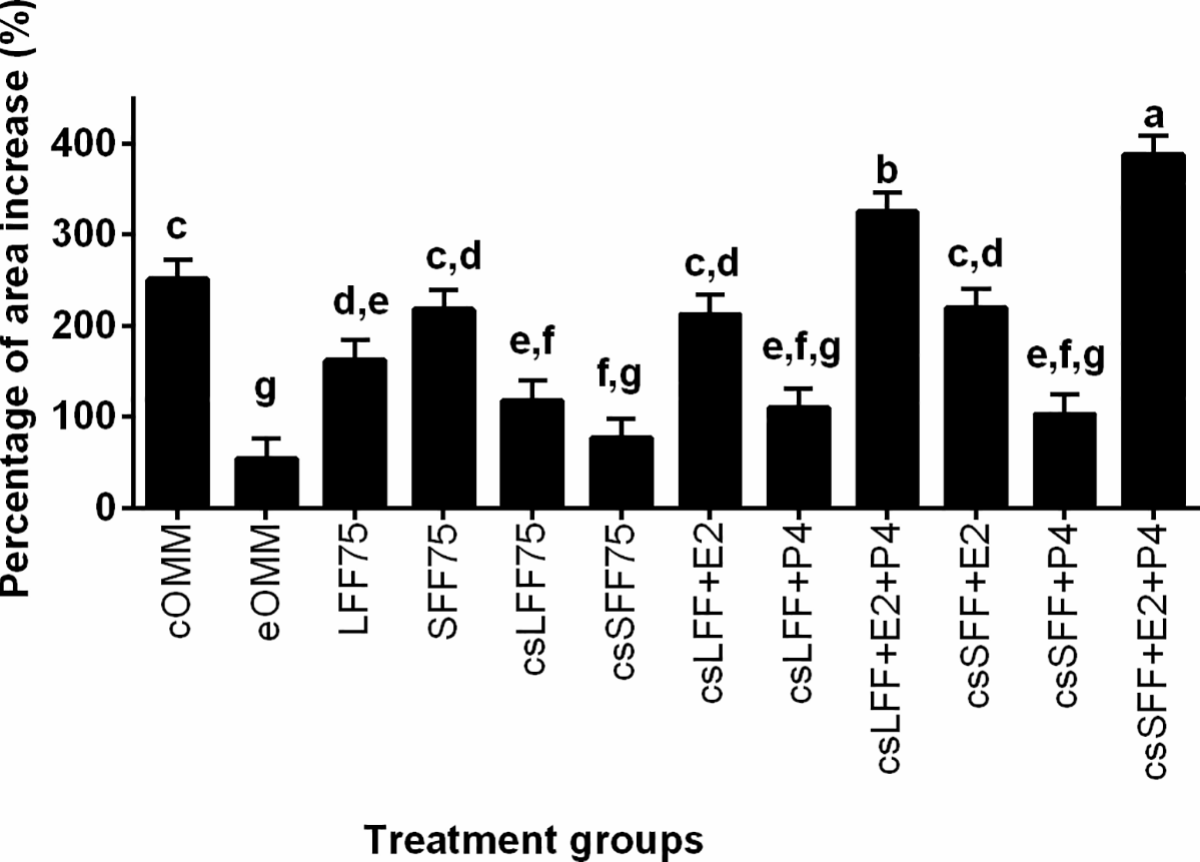
Effect of follicular fluid and steroids during the maturation period on cumulus cell expansion (percentage of area increase). Cumulus oocyte complexes (n=480) were matured in oocyte maturation medium supplemented with 0% (cOMM, eOMM) or various combinations of follicular fluid and steroids throughout the maturation period. cOMM= control oocyte maturation medium; eOMM= experimental OMM (contains charcoal stripped (cs) fetal bovine serum, no FSH, no estradiol and no EGF); LFF75= 75% untreated large follicular fluid; SFF75= 75% untreated small follicular fluid; csLFF75= 75% charcoal stripped large follicular fluid; csSFF75= 75% charcoal stripped small follicular fluid; E2= estradiol (23 ng/ml for csSFF and 37 ng/ml for csLFF treatments); P4= progesterone (140 ng/ml for csSFF and 160 ng/ml for csLFF treatments). Different letters above the bars indicate significant difference between treatments (P≤0.04).

**Fig 2:**
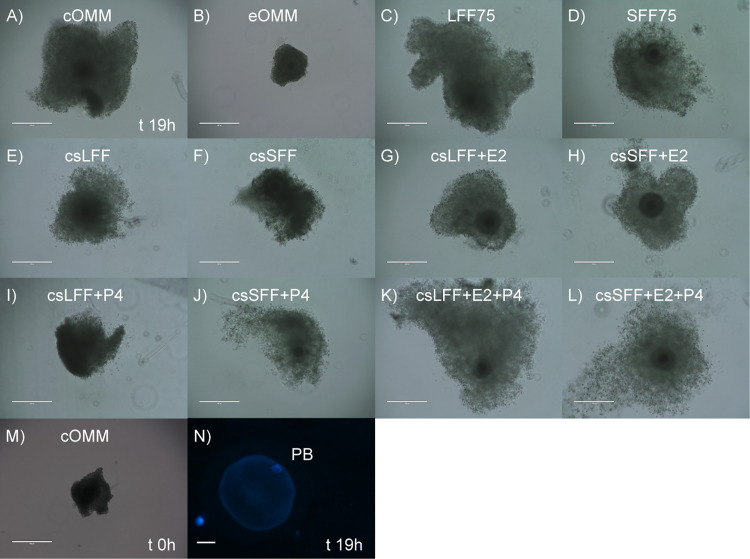
Representative image of oocyte maturation in response to exposure to follicular fluid. (A-L) Cumulus oocyte complexes (COCs) were matured for 19 h in the presence or absence of 75% follicular fluid as an additive to the oocyte maturation medium; cOMM= control oocyte maturation medium; eOMM= experimental OMM (contains charcoal stripped (cs) fetal bovine serum, no FSH, no estradiol and no EGF); LFF75= 75% untreated large follicular fluid; SFF75= 75% untreated small follicular fluid; csLFF75= 75% charcoal stripped large follicular fluid; csSFF75= 75% charcoal stripped small follicular fluid; E2= estradiol (23 ng/ml for csSFF and 37 ng/ml for csLFF treatment); P4= progesterone (140 ng/ml for csSFF and 160 ng/ml for csLFF treatment). (M) Representative COCs at the time of collection and beginning of maturation (time 0, **t** 0h). Scale bar on figures A-M = 400µm; images captured at 10X. (N) Denuded mature oocyte with the extruded polar body (PB). DNA was labeled with Hoescht 33342 (blue). There were no differences in the nuclear maturation rates as an effect of treatment. Scale bar on panel **N** = 50µm; image captured at 40X magnification.

In order to test whether estradiol and/or progesterone content of the follicular fluid was affecting cumulus cell expansion, OMM was supplemented with follicular fluid that was untreated, charcoal stripped to remove steroids, or charcoal stripped with added progesterone and/or estradiol. Table 2 summarizes the average expansion amongst all twelve treatments and Fig 1 compares the expansion percent increase from the start of maturation to the end of maturation (19 h) between treatments. Representative images of COCs at 19 h of exposure to the treatment groups are shown in Fig 2. In the absence of steroid supplementation, addition of follicular fluid to the maturation media generally reduced cumulus cell expansion in comparison to the standard OMM, and charcoal stripping further reduced expansion for the small follicular fluid treatment. In contrast, the combination of estradiol and progesterone with charcoal-stripped small follicular fluid resulted in the greatest overall increase in size of the COCs (P<0.01; Table 2 and Fig 1). Those COCs exposed to charcoal-stripped large follicular fluid with both steroids also expanded more than those matured in standard OMM (P≤0.01), but they did not achieve the same degree of expansion as those treated with charcoal-stripped small follicular fluid, estradiol and progesterone. The addition of charcoal-stripped follicular fluid (large or small) plus estradiol to maturation media resulted in cumulus cell expansion that was similar to the standard OMM. The same could not be said for supplementation with follicular fluid (large or small) plus progesterone, as this treatment negatively impacted the size (Table 2) and percentage of expansion (Fig 1) by the end of the maturation period.

**Table 2:** Area of cumulus oocyte complexes (COCs; μm^2^) at 0 and 19 h of the maturation period. Cumulus oocyte complexes (n=480) were matured in one of two control or ten follicular fluid-containing media (75% follicular fluid supplementation). Follicular fluid originated from large or small follicles and was either untreated, charcoal stripped, or charcoal stripped and supplemented with estradiol and/or progesterone. Data presented as least squares mean ± SEM (x100000).

Treatment^*^	Total COCs	0 h^**^	19 h
cOMM	40	1.77±0.17	5.99±0.42^b,c^
eOMM	40	1.62±0.18	2.19±0.43^h^
LFF75	40	1.62±0.18	4.02±0.43^e,f^
SFF75	40	1.66±0.17	4.91±0.41^c,d,e^
csLFF75	40	2.10±0.18	4.43±0.43^d,e,f^
csSFF75	40	1.72±0.17	2.85±0.41^g,h^
csLFF+E2	40	1.90±0.17	5.22±0.42^b,c,d^
csLFF+P4	40	1.68±0.17	3.42±0.41^f,g^
csLFF+E2+P4	40	1.69±0.17	6.23±0.41^b^
csSFF+E2	40	1.77±0.17	4.88±0.41^c,d,e^
csSFF+P4	40	1.86±0.17	3.68±0.41^f,g^
csSFF+E2+P4	40	1.59±0.17	7.65±0.41^a^

*cOMM= standard oocyte maturation medium; eOMM= experimental OMM (contains charcoal stripped (cs) fetal bovine serum, no FSH, no estradiol and no EGF); LFF75= large follicular fluid (>10mm); SFF75= small follicular fluid (3–5 mm); estradiol (E2) final concentration is 23 ng/ml in SFF groups and 37 ng/ml in LFF groups; progesterone (P4) final concentration is 140 ng/ml in SFF groups and 160 ng/ml in LFF groups.

**There were no differences in the average area of COCs at the onset of maturation.

^abcd^Values within row with different superscripts are significantly different (P≤0.04).

### Experiment 2: Embryo development following oocyte maturation with varying estradiol and progesterone concentrations in follicular fluid

**Fig 3:**
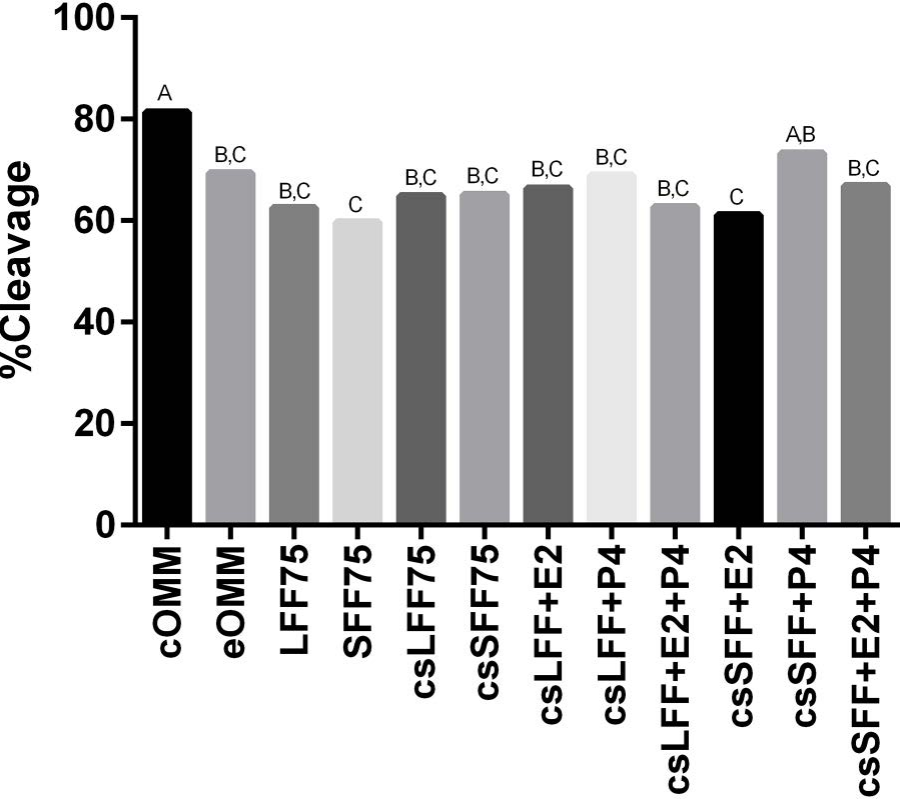
Effect of follicular fluid and steroids during the maturation period on cleavage rates. Cumulus oocyte complexes (n=4,006) were matured in oocyte maturation medium supplemented with 0% (cOMM, eOMM) or various combinations of follicular fluid and steroids throughout the maturation period. cOMM= control oocyte maturation medium; eOMM= experimental OMM (contains charcoal stripped (cs) fetal bovine serum, no FSH, no estradiol and no EGF); LFF75= 75% untreated large follicular fluid; SFF75= 75% untreated small follicular fluid; csLFF75= 75% charcoal stripped large follicular fluid; csSFF75= 75% charcoal stripped small follicular fluid; E2= estradiol (23 ng/ml for csSFF and 37 ng/ml for csLFF treatments); P4= progesterone (140 ng/ml for csSFF and 160 ng/ml for csLFF treatments). Different letters above the bars indicate significant difference between treatments (P≤0.05).

Cleavage rates were affected by treatment (Fig 3, S1 Table; P<0.01). The cleavage rate for the cOMM treatment exceeded the eOMM and all follicular fluid treatments (P<0.05) except for one: the csSFF+P4 treatment. The csSFF+P4 treatment was statistically similar to all treatments except for SFF75 and csSFF+E2, which were lower (P<0.05). Thus, of all the treatments involving follicular fluid, the only differences in cleavage rate that existed were between csSFF+P4 compared to SFF75 and csSFF+E2.

**Fig 4:**
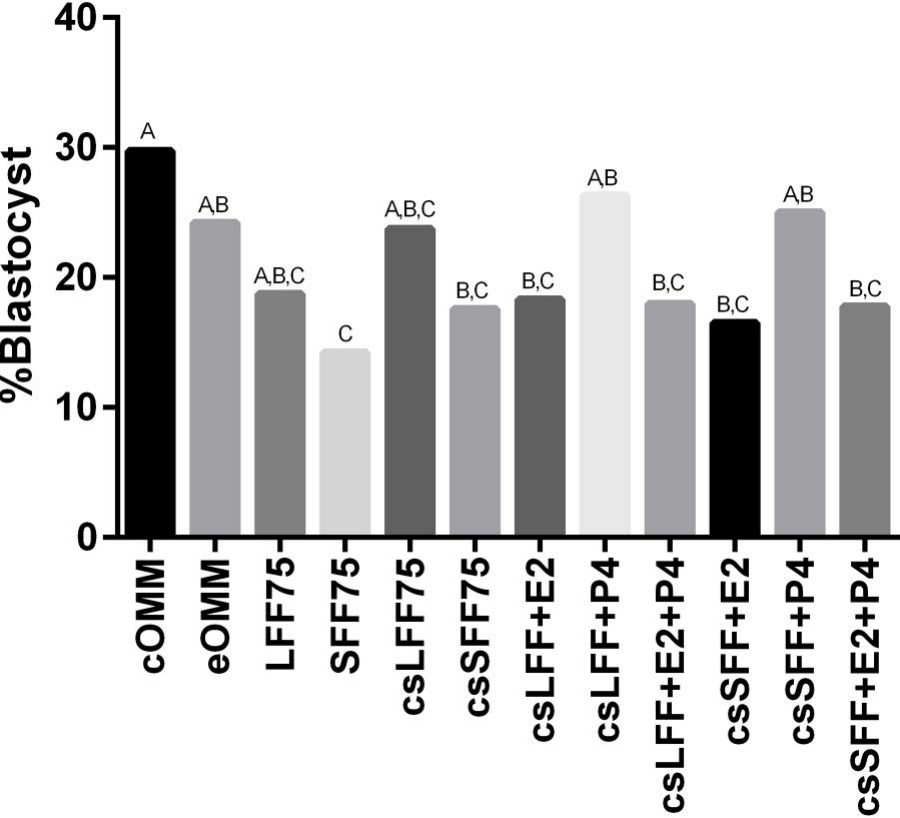
Effect of follicular fluid and steroids during the maturation period on blastocyst development. Cumulus oocyte complexes (n=4,006) were matured in oocyte maturation medium supplemented with 0% (cOMM, eOMM) or various combinations of follicular fluid and steroids throughout the maturation period. cOMM= control oocyte maturation medium; eOMM= experimental OMM (contains charcoal stripped (cs) fetal bovine serum, no FSH, no estradiol and no EGF); LFF75= 75% untreated large follicular fluid; SFF75= 75% untreated small follicular fluid; csLFF75= 75% charcoal stripped large follicular fluid; csSFF75= 75% charcoal stripped small follicular fluid; E2= estradiol (23 ng/ml for csSFF and 37 ng/ml for csLFF treatments); P4= progesterone (140 ng/ml for csSFF and 160 ng/ml for csLFF treatments). Different letters above the bars indicate significant difference between treatments (P≤0.05).

Blastocyst rates were also affected by treatment (Fig 4, S1 Table; P<0.01). As with cleavage rates, the cOMM treatment yielded the numerically highest blastocyst rate. Unlike with cleavage rates, however, there were several treatments that were statistically similar to cOMM, including eOMM, LFF75, csLFF75, csLFF+P4 and csSFF+P4. Blastocyst rates for cOMM were greater than the remaining treatments (SFF75 P<0.01; csSFF75 P<0.01; csLFF+E2 P<0.05; csLFF+E2+P4 P<0.05; csSFF+E2 P<0.01; and csSFF+E2+P4 P<0.05). On the other end of the spectrum, the blastocyst rate was numerically lowest for the SFF75 treatment. The SFF75 treatment was statistically similar to seven of the other eleven treatments, but differed from cOMM (P<0.01), eOMM (P<0.05), csLFF+P4 (P<0.01) and csSFF+P4 (P<0.05).

## Discussion

The results of this study clearly demonstrate the importance of follicular fluid-derived hormones for COC maturation. This is especially evident when comparing cumulus cell expansion and subsequent cleavage rates of the cOMM to that of the eOMM. Recall that the eOMM is devoid of steroids as it contains charcoal-stripped FBS and had no hormones or follicular fluid added. Cumulus cell expansion was severely reduced, and cleavage rates also declined when COCs were matured in this hormone-free environment. The degree of COC expansion recovered when follicular fluid was added to eOMM (fully recovered with SFF75 and partially recovered with LFF75). The observed advantage for small follicular fluid over large follicular fluid for cumulus expansion is similar to our previous findings [19]. When follicular fluid from the small follicles was then charcoal stripped, thus removing its steroid content, cumulus cell expansion again decreased to rates similar to eOMM alone.

In contrast to the results of previous work conducted by our laboratory [19], the addition of 75% follicular fluid to maturation media reduced cleavage rates in comparison to the cOMM in almost all instances. Overall, the cleavage rate for the cOMM treatment in this study was greater than our previous work, which may have contributed to the discrepancy. Some variation in IVF benchmarks (such as maturation, cleavage and blastocyst rates) between replicates and experiments is common since COCs are collected from slaughterhouse ovaries where source, age and condition of available cattle vary greatly. Under ideal conditions, follicular fluid and COCs for the current experiment would have been collected from donors of known age, physiological status and stage of the estrus cycle. Collecting from live donor animals would have presented its own challenges (such as collecting a large volume of follicular fluid from 2–5 mm follicles without blood contamination), however, and given the objectives of this experiment, the benefits would not have outweighed those challenges. Overall, cleavage rates for all treatments were acceptable at 60% or greater and were not affected by charcoal-stripping. Blastocyst rates in the current study, however, did agree with our previous results [19]. Follicular fluid added to maturation media at 75% was detrimental for blastocyst rates, especially when the fluid came from small follicles. Previous work also suggests that the overall blastocyst rates would have been greater if we had limited the follicular fluid inclusion rate to 50%. For the objectives of this study, however, we felt it was advantageous to include as much follicular fluid in the treatments as possible in order to amplify the effects of the steroid concentrations in the treatments. Charcoal stripping of the follicular fluid did not affect blastocyst rates, although it is worthwhile to note that csLFF75 was one of the few follicular fluid treatments that yielded a blastocyst rate greater than 20%.

Since charcoal-stripping indiscriminately removes steroids from the follicular fluid, the next step was to determine whether the observed differences (especially cumulus cell expansion) were specifically related to the estradiol and progesterone content of the follicular fluid. Exogenous estradiol and progesterone were added to the follicular fluid treatments at concentrations that were typical for the respective follicle size. Addition of estradiol alone returned cumulus cell expansion to control levels regardless of follicle size, while the addition of progesterone alone had no effect. The treatments that were most beneficial for cumulus cell expansion, however, were those where both estradiol and progesterone were added to the charcoal-stripped follicular fluid. Cumulus cell expansion was greatest for csSFF+E2+P4 followed by csLFF+E2+P4 and cumulus cell expansion for both exceeded that of cOMM. Cumulus cell expansion with estradiol and progesterone combined was also greater than estradiol alone and since progesterone alone had no effect on expansion, these findings suggest a synergistic relationship between these two steroids. These results agree with previously published work demonstrating the importance of optimal progesterone and estradiol concentrations in the follicular fluid for *in vivo* cumulus cell expansion [33].

Regarding cleavage rates, the addition of estradiol alone or in combination with progesterone to the maturation media had no discernible effect. The addition of progesterone to charcoal stripped-small follicular fluid improved cleavage rates such that they were similar to those of the cOMM. This beneficial effect of progesterone carried over into the resulting blastocyst rates. When comparing treatments where estradiol and progesterone were added to the follicular fluid either alone or in combination, it was the treatments with progesterone alone that stood out above all others. The csLFF+P4 and csSFF+P4 treatments achieved blastocyst rates of 25% or greater, which were similar to those of the cOMM. Unlike the cumulus cell expansion results, there were no synergistic effects on blastocyst rates from the combination of estradiol and progesterone. Blastocyst rates for the csLFF+E2+P4 and csSFF+E2+P4 treatments were less than those with progesterone alone and similar to those with estradiol alone. This suggests that the addition of estradiol to the follicular fluid maturation media is somehow suppressing the beneficial effects of progesterone.

To our knowledge this is the first study examining the effects of follicular fluid supplementation in maturation media in relation to follicle size with or without the addition of estradiol and progesterone on COC expansion and subsequent early embryonic development. The addition of the follicular fluid treatments to the maturation media had variable effects on COC expansion, while it either maintained or reduced cleavage and blastocyst rates in comparison to the cOMM treatment. Regarding the addition of progesterone and estradiol to the maturation media, it is important to note that we did not monitor fluctuations of either of these steroids during the maturation phase. Concentrations of estradiol and progesterone would have undoubtedly fluctuated in a dynamic fashion due to factors such as leaching of steroids into the mineral oil overlay and cumulus cell production of steroids [34–36]. Even though we are unable to report the concentrations of estradiol and progesterone throughout the maturation phase, we do know the initial concentrations of these hormones in the maturation media. Those specific concentrations may have only been maintained for the first few hours of maturation [35], but were sufficient enough to cause treatment-based differences in our outcomes of interest.

The effects of the treatments with added estradiol and/or progesterone suggest a dichotomous relationship between these two follicular steroid hormones. Estradiol is beneficial for cumulus cell expansion, and thus presumably oocyte maturation [37], as the addition of estradiol to the charcoal stripped follicular fluid treatments improved cumulus cell expansion over that of progesterone alone. Furthermore, the combination of estradiol and progesterone produced a synergistic benefit for cumulus cell expansion. The fact that the combination treatment that included small follicular fluid outperformed that of its counterpart with large follicular fluid further emphasizes the importance of estradiol for cumulus cell expansion as the estradiol:progesterone ratio is greater in the treatments with small follicular fluid. While estradiol was beneficial for cumulus cell expansion, it was the addition of progesterone to charcoal-stripped follicular fluid treatments that improved blastocyst rates. This finding is consistent with previously published work that reported the importance of progesterone during *in vitro* maturation for oocyte developmental competence [38], although results of other studies have been mixed [3, 38–40]. In the current study, the progesterone-related improvement was evident in treatments containing follicular fluid from both small and large follicles. Interestingly, the treatments with both estradiol and progesterone did not promote blastocyst development, further emphasizing the importance of progesterone and suggesting that estradiol negates the positive effects of progesterone.

Estradiol is commonly included in maturation media formulations for bovine oocytes in conjunction with FSH. When estradiol is added to maturation media in the absence of gonadotropins, blastocyst rates are reduced [41]. This effect is either reversed or attenuated when estradiol is added to maturation media alongside LH or FSH [21, 41]. Gonadotropins would have presumably been present in the follicular fluid used in the treatments of the current study as they would not have been removed by charcoal stripping, yet blastocyst development was not improved with estradiol treatment. It is important to note that progesterone supplementation of the maturation media was not tested alongside estradiol supplementation in the previous work and neither of these experiments included follicular fluid in the maturation media [21, 41].

Fetal bovine serum was used in the control maturation media formulations in the current experiment (cOMM included non-charcoal-stripped FBS, eOMM contained charcoal-stripped FBS). It contains a number of factors that can affect the COCs, including hormones, growth factors, proteins, extracellular vesicles, carbohydrates and lipids [42, 43], and it is commonly included in maturation media and other cell culture media formulations. In the current study, when steroids were removed from the FBS by charcoal stripping, cumulus cell expansion was severely reduced. By the time the COCs progressed through the IVF procedures and were evaluated for blastocyst rates, however, the difference between those matured in the cOMM and eOMM disappeared. These findings suggest that even though the lack of steroids in the eOMM was clearly detrimental for cumulus cell expansion, the COCs can rebound and produce viable embryos.

Considering that the COCs in the current study were only exposed to the follicular fluid treatments during the maturation phase of *in vitro* embryo production, it is somewhat surprising to find that the treatments that benefitted cumulus cell expansion did not also support blastocyst development. One would think that improved cumulus cell expansion (and thus, estradiol treatment of the maturation media) would likely be related to greater oocyte competence, which would ultimately increase blastocyst rates [33]. This was not the case, however, as the degree of expansion of the COCs did not seem to influence subsequent blastocyst rates. Instead, it was the addition of progesterone to the follicular fluid treatments during the maturation phase that improved blastocyst rates. During *in vivo* development, the oocyte undergoes maturation while bathed in follicular fluid under the influence of decreasing estradiol and increasing progesterone [39, 44–46]. The results of this study highlight the importance of this transition, as progesterone exposure during maturation appears to support blastocyst development.

## Conclusions

Taken together, the results of these experiments demonstrate that estradiol exposure during the maturation phase is beneficial for cumulus cell expansion (small follicular fluid and supplementation with estradiol) and that estradiol and progesterone can function synergistically to further increase cumulus cell expansion. For blastocyst rates, however, it was the progesterone-enriched environment during the maturation phase that was most beneficial (large follicular fluid and supplementation with progesterone). In this study, follicular fluid was added to the treatments at a relatively high inclusion rate of 75%, which left opportunity for other undefined proteins or factors in the maturation media to overshadow the effects of the added steroids. While there was some minor evidence for non-steroidal influence on cumulus cell expansion and blastocyst rates, the consistency of the results from estradiol and/or progesterone addition to the maturation media highlights the overwhelming importance of these hormones for oocyte developmental competence.

## Supporting information

S1 Table Effect of follicular fluid and steroids during the maturation period on cleavage and blastocyst rates. (DOCX)
