# Supplementary material for: Maturation of bovine cumulus oocyte complexes in follicular fluid with or without estradiol, progesterone or the combination affects cumulus cell expansion and blastocyst development
Source: PLoS One. 2025 Jun 2;20(6):e0321266. doi: 10.1371/journal.pone.0321266 (PMC12129229; doi:10.1371/journal.pone.0321266)
Supplement: S1 Table — (DOCX) [file pone.0321266.s001.docx]

| **S1 Table.** Effect of follicular fluid and steroids during the maturation period on cleavage and blastocyst rates. Cumulus oocyte complexes (n=4,006) were matured in oocyte maturation medium supplemented with 0% (cOMM, eOMM) or various combinations of follicular fluid and steroids throughout the maturation period. cOMM= control oocyte maturation medium; eOMM= experimental OMM (contains charcoal stripped (cs) fetal bovine serum, no FSH, no estradiol and no EGF); LFF75= 75% untreated large follicular fluid; SFF75= 75% untreated small follicular fluid; csLFF75= 75% charcoal stripped large follicular fluid; csSFF75= 75% charcoal stripped small follicular fluid; E2= estradiol (23 ng/ml for csSFF and 37 ng/ml for csLFF treatments); P4= progesterone (140 ng/ml for csSFF and 160 ng/ml for csLFF treatments). | | | |
| --- | --- | --- | --- |
| Treatment^*^ | Total COCs | Cleavage Rates | Blastocyst Rates** |
| cOMM | 504 | 81.35 | 29.76 |
| eOMM | 392 | 69.39 | 24.23 |
| LFF75 | 240 | 62.50 | 18.75 |
| SFF75 | 372 | 59.68 | 14.25 |
| csLFF75 | 282 | 64.89 | 23.76 |
| csSFF75 | 364 | 65.11 | 17.58 |
| csLFF+E2 | 262 | 66.41 | 18.32 |
| csLFF+P4 | 319 | 68.97 | 26.33 |
| csLFF+E2+P4 | 295 | 62.71 | 17.97 |
| csSFF+E2 | 345 | 61.16 | 16.52 |
| csSFF+P4 | 344 | 73.26 | 25.00 |
| csSFF+E2+P4 | 287 | 66.90 | 17.77 |
| ^*^cOMM= standard oocyte maturation medium; eOMM= experimental OMM (contains charcoal stripped (cs) fetal bovine serum, no FSH, no estradiol and no EGF); LFF75= large follicular fluid (>10mm); SFF75= small follicular fluid (3-5mm); estradiol (E2) final concentration is 23 ng/ml in SFF groups and 37 ng/ml in LFF groups; progesterone (P4) final concentration is 140 ng/ml in SFF groups and 160 ng/ml in LFF groups.  ^**^Day 8 blastocyst rate was calculated as the total number of blastocysts divided by the total number cleaved. | | | |
